# Advances in Anti-obesity Pharmacotherapy: Current Treatments, Emerging Therapies, and Challenges

**DOI:** 10.7759/cureus.46623

**Published:** 2023-10-07

**Authors:** Skyler Brandfon, Adi Eylon, Deepesh Khanna, Mayur S Parmar

**Affiliations:** 1 Osteopathic Medicine, Nova Southeastern University Dr. Kiran C. Patel College of Osteopathic Medicine, Fort Lauderdale, USA; 2 Foundational Sciences, Nova Southeastern University Dr. Kiran C. Patel College of Osteopathic Medicine, Clearwater, USA

**Keywords:** obesity-related illnesses, mc4r agonists, leptin analogue, lipase inhibitors, glp-1 receptor agonists, anti-obesity medications, overweight and obesity

## Abstract

Obesity is a major public health concern linked to health risks such as hypertension, hyperlipidemia, type 2 diabetes mellitus (T2DM), stroke, metabolic syndrome, asthma, and cancer. It is among the leading causes of morbidity and mortality worldwide caused by an unhealthy diet and lack of physical activity, but genetic or hormonal factors may also contribute. Over a third of adults in the United States are obese. Pharmacological agents have been designed to reduce weight gain caused by excessive calorie intake and low physical activity. They work by inhibiting the absorption of dietary fat or stimulating the secretion of satiety hormones. These drugs include lipase inhibitors and glucagon-like peptide 1 (GLP-1) receptor agonists. However, the current weight-loss strategies do not effectively treat genetic-related diseases, such as generalized lipodystrophy, Bardet-Biedl syndrome, and proopiomelanocortin (POMC) deficiency. Emerging therapies for these gene mutations have been developed targeting leptin and melanocortin-4 receptors (MC4Rs), restoring the normal function of leptin or melanocortin-4 receptors regulating energy balance and appetite. Leptin analogs and MC4R agonists are novel therapies that target genetic or hormonal causes of obesity. This article provides a comprehensive review of anti-obesity medications (AOMs). In this review, we discuss the clinical trials, efficacy, United States FDA-approved indication, contraindications, and serious side effects of different classes of drugs, including lipase inhibitors, GLP-1 agonists, leptin analogs, and MC4R agonists.

## Introduction and background

Obesity, clinically defined as a BMI greater than 30, is the excessive accumulation of fat that poses significant risks to a person’s health. Obesity is a longstanding issue for adults and children in the United States and globally. Approximately 81% of Americans are obese, with a BMI of 30 or greater [1]. According to WHO, in 2016, over 340 million children and adolescents aged 5-19 were overweight or obese; in 2020, 39 million children under the age of five were overweight or obese. With the high prevalence of obesity comes a high mortality rate, as nearly 3 million people die yearly from being overweight or obese. Obesity significantly increases the risk for chronic health conditions, such as hypertension, hyperlipidemia, type 2 diabetes mellitus (T2DM), metabolic syndrome, sleep apnea, asthma, osteoarthritis, and gallbladder disease [2-7]. It is also associated with an increased risk for stroke, depression, anxiety, and certain cancers (including those related to the breast, colon/rectum, gallbladder, stomach, liver, kidneys, ovaries, pancreas, and thyroid) [8,9]. Risk factors for obesity include a lack of physical activity and an unhealthy diet of excess calories, saturated fat, and sugar. Other factors that may promote obesity are lack of quality sleep, stress, certain health conditions, genetics, medications, and one’s environment.

The balance of energy intake and expenditure determines obesity. The overconsumption of calories without the proper means to burn them results in weight gain, and, in the absence of adequate modification of lifestyle, diet, and exercise, can gradually result in obesity. Weight loss occurs typically when caloric intake is less than its expenditure. The combination of diet and aerobic exercise training has been proven to result in weight loss and provide long-term health benefits, such as reducing the risk of diabetes, heart disease, hypertension, stroke, and some cancers. However, individuals with various health conditions associated with hormonal imbalances and genetic mutations find it challenging to lose weight simply through these non-pharmacological approaches, further contributing to the need for pharmacological agents to treat them. For instance, insulin resistance is seen in T2DM and polycystic ovary syndrome (PCOS), characterized by the body’s weak response to insulin and the consequential need for the pancreas to produce more insulin to maintain healthy blood glucose levels. Elevated insulin levels promote excess fat storage, and weight gain occurs, thus making insulin resistance worse [10]. In hypothyroidism, the lack of thyroid hormone results in a slower metabolism, making it hard to lose weight. Furthermore, genetic mutations in the hormones and proteins responsible for regulating hunger and satiety make obesity inevitable. In proopiomelanocortin (POMC) deficiency, there is decreased stimulation of melanocortin-4 receptor (MC4R) in the CNS, resulting in reduced satiety and lack of energy regulation.

The longstanding difficulty for many individuals to effectively manage their obesity due to external or internal factors has emphasized the need for pharmacological interventions. Thus, the prevalence and severity of the obesity epidemic have resulted in various drug developments targeted to reduce appetite and aid in weight loss. This review article summarizes the clinical trials, mechanism of action, and adverse effects of each class of anti-obesity medications (AOMs). It provides suggestions for their potential uses in obesity management.

## Review

Primary approaches to managing obesity include behavioral modification, such as eating a balanced diet on a weight-dependent caloric deficit and participating in 30 to 60 minutes of daily physical activity [11]. Another non-pharmacological strategy for weight loss is cognitive behavioral therapy to overcome emotional and psychological barriers to controlling weight [12,13]. Complementary approaches clinically shown to decrease body weight (albeit with limited supportive data) include acupuncture, vitamin D supplementation, and N-3 fatty acids (EPA + DHA) supplementation [14]. Bariatric surgery, such as gastric bypass surgery and sleeve gastrectomy, is a more invasive non-pharmacological last resort approach that successfully treats obesity by physically restricting the amount of food an individual can consume [14].

**Figure 1:**
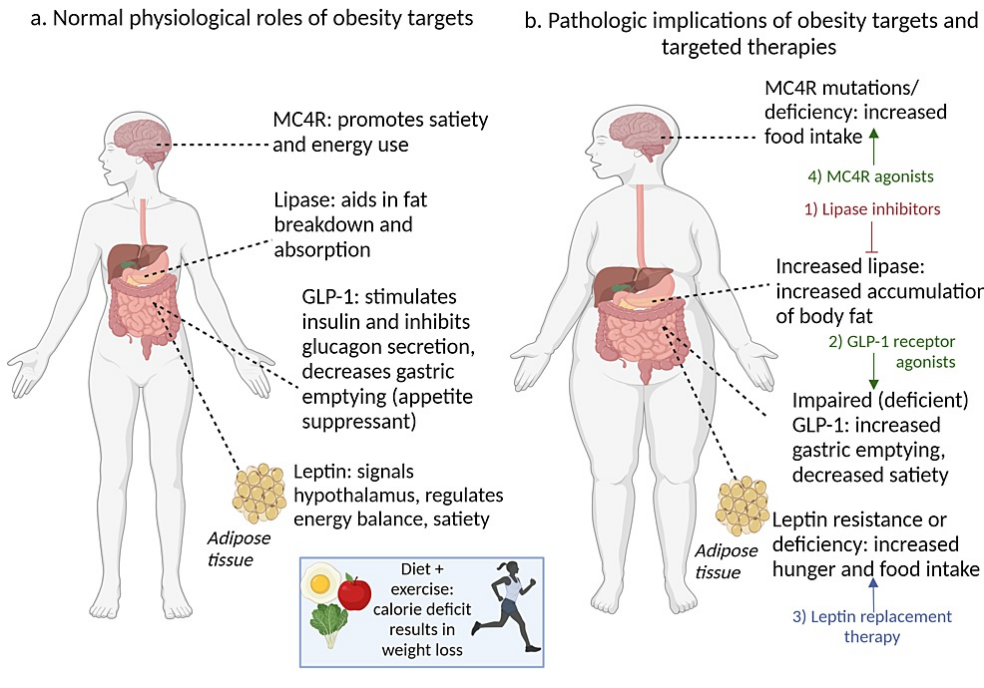
The physiological and pathological roles of obesity targets and anti-obesity therapies (A) The normal physiological role of obesity targets. (B) Pathological implications of obesity targets and anti-obesity therapies. The illustration was created using BioRender.com. Image credit: Adi Eylon.

Pharmacological approaches include targeting enzymes and hormones implicated in the pathophysiology of obesity. These targets include lipase (an enzyme involved in fat breakdown and absorption), glucagon-like peptide 1 (GLP-1; a hormone that promotes postprandial satiety), leptin (a key regulator of hunger), and MC4R (a G-protein coupled receptor that regulates energy homeostasis). Figure 1 illustrates the physiological and pathological roles of obesity targets and anti-obesity therapies.

Lipase inhibitors

In its naturally occurring state, lipoprotein lipase is a versatile enzyme that digests fats and lipids, hydrolyzes fats into fatty acids and glycerol, increases fat absorption, and maintains gallbladder function. Its favorability toward fat breakdown and absorption makes lipase a significant contributor to obesity. Lipase's function is to remove triglycerides from circulation and increase fat absorption; thus, body fat accumulates from the distribution of hydrolyzed triglycerides. This genetic role of lipase has made possible the development of lipase inhibitor drugs, orlistat and cetilistat, for weight loss in obese patients. Orlistat was FDA-approved as over-the-counter in 2007 for weight loss in obese patients, while cetilistat is not yet FDA-approved in the United States. Cetilistat was approved in Japan in 2013 for weight loss in obese patients with comorbidities [15].

Drug Development and Mechanisms of Action

By isolating a saturated derivative of endogenous lipstatin from *Streptomyces toxytricini*, a gram-positive bacterium, the formation of orlistat was found to have a naturally inhibiting effect on lipoprotein lipase. Orlistat acts by reversibly inhibiting lipases by covalently binding to serine residues of active sites and inactivating them, thereby blocking fat hydrolysis and decreasing fat absorption. Acting locally via the intestinal wall, the benefits of orlistat include blood pressure improvement and weight loss, thereby reducing the incidence of T2DM, as was found in the XENDOS clinical study [15]. Although orlistat is effective in prediabetic patients, it lacks a role in decreasing obesity in patients diagnosed with DM. The reasoning behind this is unknown, and this unassurance led to the creation of a novel drug. Table 1 describes the up-to-date information about FDA-approval, drug dosing, indications, contraindications, and serious adverse effects of lipase inhibitors.

**Table 1:** Lipase Inhibitors as AOMs: FDA-approval, drug dosing, indications, contraindications, and serious adverse effects

Drug	Formulation/dosing	Contraindications	Possible serious adverse effects	FDA-approval (year and indications)
					Orlistat (Xenical^®^)	Oral: One 120-mg capsule three times a day with each main meal containing fat (during or up to 1 hour after the meal)	Pregnancy	Oily spotting	Approved in 1999
Known hypersensitivity to orlistat or to any component of this product	Flatus with discharge and fecal urgency	Used for obesity management, including weight loss and maintenance, when used with a reduced-calorie diet				
Chronic malabsorption syndrome	Fatty/oily stool and oily evacuation	Also indicated to reduction in the risk for weight regain after prior weight loss				
Cholestasis	Increased defecation and fecal incontinence					
Cetilistat (Oblean^®^)	No information available	Known hypersensitivity to cetilistat	Fecal incontinence	Not approved by the FDA in the USA
			Rectal discharge and defecation urgency	Approved in Japan in 2013

Alizyme and Takeda Pharmaceuticals developed cetilistat, an oral, highly lipophilic benzoxazinone lipase inhibitor. Cetilistat has not been approved by the FDA yet, but it has shown promising results in a clinical trial and has the potential to be a safe and effective weight-loss drug. A 12-week randomized, placebo-controlled study found that cetilistat produced significant weight loss and other obesity-related parameters in obese patients [16]. Another phase 2 trial compared three doses of cetilistat (40, 80, and 120 mg three times a day) with a placebo for 12 weeks in obese patients with T2DM who were taking metformin. The results showed that cetilistat 80 or 120 mg reduced body weight and improved blood sugar control significantly more than placebo [17]. Cetilistat had fewer gastrointestinal side effects than orlistat [17]. The reasoning behind this difference is not clear. Cetilistat and orlistat have similar mechanisms of action but have different chemical structures that may affect how they interact with fat in the intestine. Cetilistat may have some drawbacks, such as malabsorption of nutrients and vitamins that need fat to be absorbed. Cetilistat has effectively reduced total serum cholesterol and LDL cholesterol in obese patients and glycosylated hemoglobin, HbA1c, in obese diabetics [18]. Cetilistat and orlistat are meant to be used with a low-calorie, low-fat diet for optimal weight loss. Table 1 describes the up-to-date information about FDA-approval, drug dosing, indications, contraindications, and serious adverse effects of lipase inhibitors.

GLP-1 receptor agonists

GLP-1 is vital to the human endocrine system, a peptide hormone produced by intestinal epithelial endocrine cells in response to meal intake [19]. GLP-1 has multiple functions contributing to regulating blood glucose levels and weight maintenance. GLP-1 stimulates insulin secretion and inhibits glucagon secretion, thereby playing a role in limiting postprandial glucose accumulation. Pathological excess of GLP-1 contributes to hypoglycemia, while pathological deficiency contributes to obesity [20]. Regarding weight maintenance, GLP-1 functions as a natural appetite suppressant because it decreases gastric emptying and intestinal motility, signaling a longer amount of postprandial satiety. It is part of the "ileal break," a negative feedback mechanism optimizing nutrient absorption and digestion [21].

Endogenous GLP-1 is broken down at position 2 (alanine) by dipeptidyl peptidase-4 (DDP-4) and has too short of a half-life (1.5 hours) to be viable as a drug development for pharmacologic use. However, the receptor of GLP-1 does not get degraded by DDP-4, making GLP-1 receptor agonists a viable therapeutic option [22], leading to the development and approval of several GLP-1 receptor agonists. Current GLP-1 receptor agonists were initially designed to treat T2DM because they stimulate insulin secretion and reduce blood glucose. However, clinical observations and clinical studies pointed out their role in regulating weight management, leading to their approval for obesity. Recently, many GLP-1 receptor agonists, early designed for T2DM management, have received approval for obesity treatment.

Drug Development and Mechanisms of Action

In this section, the information about GLP-1 receptor agonists is more focused on their role in weight management and approval as AOMs; hence, no detailed information about their antidiabetic indication is covered.

The peptide agonist exendin-4 is resistant to dipeptidyl peptidase 4 (DPP-4) degradation [23] due to the second amino acid residue in the N-terminal region, which is the site of DPP-mediated inactivation (alanine for human GLP-1), being replaced by glycine in exendin-4. Exendin-4 was originally found in the lizard Heloderma suspectum. In animal models and subsequent phase 3 clinical trials, exendin-4 resulted in weight loss [24,25]. A synthetic GLP-1 receptor agonist that mimics exendin-4 was created and branded as exenatide in 2005 to create a pharmacological drug. It was the first FDA-approved GLP-1 receptor agonist for T2DM. Exenatide works by increasing glucose-dependent insulin secretion from pancreatic beta cells, decreasing glucagon secretion, delaying gastric emptying, increasing satiety, and reducing food intake. These actions are mediated by the drug binding to the pancreatic GLP-1 receptors [26]. Although some studies have shown exenatide promotes weight loss, it is not currently FDA-approved specifically for obesity treatment.

Following subcutaneous administration of exenatide, its half-life is approximately 2.5 hours; therefore, it is given twice daily [27]. To increase the duration and effectiveness of the drug, a different GLP-1 receptor agonist with a longer half-life than exenatide was highly sought, leading to the development of liraglutide. The FDA-approved it for the treatment of T2DM in 2010. Liraglutide was also approved by the FDA under the brand name Saxenda® in 2014 for chronic weight management in adults and again in 2020 for chronic weight management among pediatric patients aged 12 and older who are obese. Liraglutide exceeds exenatide's half-life of 2.5 hours at a significantly increased duration of 13 hours. It accomplishes this by having one amino acid substitution from GLP-1 (Lys34Arg) and a 16-carbon palmitic acid side chain. The N-terminus of the GLP-1 peptide sequence was modified to prevent DPP-4 degradation, and fatty acid side chains were attached to strengthen protein binding and increase half-life [22]. In phase 3 trials, subcutaneous injections of 3 mg of liraglutide once daily resulted in significant weight loss [28]. These encouraging clinical findings led to the approval of liraglutide FDA-approval under the brand name Saxenda® in 2012 for chronic weight management in adults with a BMI of 30 kg/m^2^ or higher or BMI of 27 kg/m^2^ or higher who have at least one weight-related condition. Later, in 2020, it was reapproved again for chronic weight management among pediatric patients aged 12 and older who are obese. Some of the reported adverse effects of liraglutide are primarily mild to moderate nausea and diarrhea. Despite the successful efficacy of liraglutide in T2DM and obesity management, the discomfort of once-daily injection posed a barrier for patients, thereby leading to the design of a once-weekly injectable GLP-1 receptor agonist, semaglutide [29].

In 2017, injectable semaglutide (Ozempic®) was FDA-approved for T2DM, and in 2021, it was FDA-approved for weight loss under the brand name Wegovy® [30]. A phase 2 clinical study was conducted to compare the efficacy and safety of once-daily subcutaneous (3.0 mg; initiated at 0.6 mg per day and escalated by 0.6 mg per week) liraglutide compared with once-weekly subcutaneous (0.05 mg, 0.1 mg, 0.2 mg, 0.3 mg, or 0.4 mg; initiated at 0.05 mg per day and incrementally escalated every four weeks) semaglutide, added to counseling for diet and physical activity, or weight loss in patients with obesity without diabetes. The results showed that subcutaneous semaglutide significantly reduced mean body weight compared to subcutaneous liraglutide [31]. However, both drugs were effective for weight loss [31]. The clinical trial results that led to approval demonstrated that subcutaneous injection of 2.4 mg of semaglutide once weekly plus lifestyle intervention was associated with sustained, clinically relevant reduction in body weight with mild adverse effects similar to those of liraglutide [32,33]. A randomized, open-label, 68-week, phase 3b trial was also conducted to compare the efficacy and adverse event profiles of once-weekly subcutaneous (2.4 mg) semaglutide compared to once-daily subcutaneous (3.0 mg) liraglutide (both with diet and physical activity) in people with overweight or obesity without diabetes [34]. The mean body weight change from baseline was higher with semaglutide as participants had significantly greater odds of achieving 10% or more, 15% or more, and 20% or more weight loss with semaglutide vs. liraglutide [34]. Gastrointestinal adverse effect was most noted with both these drugs [34]. In regular clinical settings, semaglutide treatment (1.7 mg or 2.4 mg for three to six months) was also associated with weight loss, like observations from randomized clinical trials [35].

Wegovy® and Ozempic® are both semaglutide, but they differ in dosing. Wegovy is available in higher doses (2.4 mg, once weekly), and Ozempic® is offered in 0.5, 1.0, and 2.0 mg doses once weekly. Unlike Wegovy®, Ozempic® has been indicated for treating T2DM only since its FDA-approval in 2017, while Wegovy® is FDA-approved only for chronic weight management in adults with obesity or overweight with at least one weight-related condition, such as high blood pressure, T2DM, or high cholesterol. Table 2 describes the up-to-date information about FDA-approval, drug dosing, indications, contraindications, and serious adverse effects of GLP-1 receptor agonists.

**Table 2:** GLP-1 receptor agonists as AOMs: FDA-approval, drug dosing, indications, contraindications, and serious adverse effects

Drug	Formulation/dosing	Contraindications	Possible serious adverse effects	FDA-approval (year and indications)
					Liraglutide (Saxenda^®^)	Injection (subcutaneous): 3 mg of liraglutide injection once daily, with or without food. The dose should be increased gradually from 0.6 mg to 3 mg over 5 weeks	Pregnancy	Possible thyroid tumors, including cancer	2014
History of medullary thyroid carcinoma (MTC) or multiple endocrine neoplasia syndrome type 2 (MEN 2)	Pancreatitis	Chronic weight management				
Acute pancreatitis	Gall bladder problem	May help adults with excess weight (BMI ≥27) who also have weight-related medical problems or obesity (BMI ≥30) and children aged 12-17 years with body weight above 132 pounds (60 kg) and obesity to help them lose weight and keep the weight off				
	Increase heart rate					
	Hypoglycemia (in adults with T2DM who also take other antidiabetic medication)					
	Serious allergic reactions					
	Depression or suicidal thoughts					
Semaglutide (Wegovy^®^)	Injection (subcutaneous): 2.4 mg of semaglutide injection once weekly, with or without food. The dose should be increased gradually from 0.25 mg to 2.4 mg over 16 weeks	Pregnancy	Possible thyroid tumors, including cancer	2021
		History of medullary thyroid carcinoma (MTC) or multiple endocrine neoplasia syndrome type 2 (MEN 2)	Pancreatitis	Chronic weight management
		Acute pancreatitis	Gall bladder problem	May help adults and children aged ≥12 years with obesity (BMI ≥30 for adults, BMI ≥95th percentile for age and sex for children)
			Increase heart rate	May help some adults with excess weight (BMI ≥27) (overweight) who also have weight-related medical problems to help them lose weight and keep it off
			Hypoglycemia (in adults with T2DM who also take other antidiabetic medication)	It should be used with a reduced calorie meal plan and increased physical activity pounds (60 kg) and obesity to help them lose weight and keep the weight off
			Serious allergic reactions	
			Depression or suicidal thoughts	

Leptin analogs

When examining the relationship between genetics and obesity, an adipocyte-secreted protein known as leptin plays a role in nutrient homeostasis and satiety; it is also known as the "satiety hormone" as it helps to signal to the brain that the body is full. Leptin is primarily known for regulating energy balance and hunger; therefore, being a critical factor in weight maintenance. Mutations in the gene that codes for leptin can lead to leptin deficiency. This can cause obesity, as the body cannot properly regulate its appetite and energy expenditure. The ob/ob mouse is a strain of mouse that has a homozygous mutation in the leptin gene. These mice are severely obese and closely resemble morbidly obese humans [36]. The discovery of the leptin gene and its role in obesity has led to the development of leptin therapy. It is important to note that leptin therapy is not FDA-approved for obesity management. However, rare cases of familial obesity due to congenital leptin deficiency have been treated successfully with exogenous leptin administration, significantly reducing hyperphagia. The challenge and non-effectiveness of leptin therapy approach in obesity management is that plasma levels of leptin are already higher than in lean ones, suggesting leptin resistance; thus, exogenous administration of leptin has little to no effect in inhibiting food intake. The mode of resistance is discussed in detail in the below section.

Drug Development and Mechanisms of Action

Leptin's mechanism of action consists of binding to leptin receptors (ObRs) in the brain and peripheral tissue. As a result of alternative splicing, ObR is transformed into an ObRa isoform, which can cross the blood-brain barrier, resulting in heavy expression in the hypothalamus. Leptin binding to its receptor activates janus kinase-signal transducer (JAK2-STAT3), which is vital to the regulation of homeostasis, as well as phosphatidylinositol 3-kinase (PI3K), which regulates food intake and glucose homeostasis [37]. If leptin is elevated, a hypothalamic negative feedback loop is activated, decreasing food intake and increasing energy expenditure [38]. Leptin's critical role as a signal-transducing cytokine and its known hypothalamic negative feedback loop are reasons for its potential in treating obesity [37,38]. However, leptin's tendency for resistance has caused reluctance in developing effective leptin analogs for treating obesity. Leptin resistance is hypothesized to occur from various mechanisms, including impairment in leptin transportation. Short forms of LEPRa and LEPRe, also known as ObRa discussed above, are thought to mediate leptin transport across the blood-brain barrier but are impaired in both obese humans and obese mice [39]. Another proposed argument for leptin resistance is the overexpression of SOCS3, the protein tyrosine phosphatase responsible for regulating leptin's negative feedback system. SOCS3 inhibits JAK2 kinase, and when overexpressed, no leptin will be produced due to a secondary defect in the feedback loop. Other possible contributors to leptin resistance include hyperleptinemia, inflammation, defective autophagy, and endoplasmic reticulum stress. Leptin's multiple mechanisms of resistance make leptin less of an ideal target the reason why there are no drugs on the market for the treatment of diet-based or externally derived obesity. However, congenital and genetic defects of leptin result in obesity from internal mechanisms. A lack of leptin contributes to congenital/acquired generalized lipodystrophy, making leptin an ideal direct drug target for this scenario in particular.

Metreleptin is a leptin replacement therapy that was FDA-approved in 2014 as an adjunct to diet, which was developed specifically for treating patients with generalized lipodystrophy [40]. Metreleptin, administered by injection, is a recombinant human leptin analog similar to endogenous leptin but has an amino-terminal methionine residue [41] that binds to the leptin receptor ObR, inducing a conformational change and activating the receptor in the same mechanism that endogenous leptin does. Metreleptin is contraindicated in patients with general, nongenetic obesity that does not result from internal leptin defects because externally derived obesity is unrelated to a leptin problem, rendering leptin analogs resistant for these patients. Metreleptin, therefore, bypasses the possibility of leptin resistance due to its indication for patients only with genetic defects in leptin who lack central leptin resistance, a type of resistance seen in nongenetic obesity. In conclusion, leptin is not the ideal drug target for treating obesity unless indicated specifically for congenital/acquired generalized lipodystrophy. Table 3 describes the up-to-date information about drug dosing, indications, contraindications, and serious adverse effects of leptin analog that is FDA-approved for leptin deficiency in patients with generalized lipodystrophy.

**Table 3:** Leptin analogs: FDA-approval, drug dosing, indications, contraindications, and serious adverse effects

Drug	Formulation/dosing	Contraindications	Possible serious adverse effects	FDA-approval (year and indications)
					Metreleptin (Myalept^®^)	Injection (subcutaneous): body weight 40 kg or less: starting dose 0.06 mg/kg/day, increase or decrease by 0.02 mg/kg to a maximum daily dose of 0.13 mg/kg	Generalized obesity not associated with leptin deficiency	Neutralizing antibodies	2014
Increased risk of lymphoma	Leptin deficiency in patients with generalized lipodystrophy						
Injection (subcutaneous): males greater than 40 kg body weight: starting dose 2.5 mg/day, increase or decrease by 1.25 mg to 2.5 mg/day to a maximum 10 mg/day dose		Hypoglycemia (if taken with other antidiabetic mediation)					
			Injection (subcutaneous): females greater than 40 kg body weight: starting dose 5 mg/day, increase or decrease by 1.25 mg to 2.5 mg/day to a maximum dose of 10 mg/day				

MC4R agonists

The MC4R is a G protein-coupled receptor encoded by the MC4R gene. MC4R was initially cloned through degenerate polymerase chain reaction (PCR) in 1993; however, MC4R's function in energy homeostasis regulation was only discovered in studies a few years later. In 1997, the hypothesized function of this receptor was validated in mice studies, and in 1998, human genetic studies revealed that MC4R genetic mutations could lead to monogenic obesity [42]. MC4R mutations are currently the leading monogenic cause of obesity. Over 150 mutations, including frameshift, nonsense, and missense, in the MC4R gene are implicated in obesity. The summation of these genetic mutations leads to an overall decrease in MC4R function, thus leading to obesity [41]. MC4R agonistic action stimulates an appetite-reducing effect and is an ideal therapeutic target in individuals with obesity (who have MC4R gene mutations) [43].

Drug Development and Mechanisms of Action

The MC4R is located in the CNS, stimulated by melanocyte-stimulating hormone, α-MSH, via depolarization and antagonized by agouti-related peptide (AgRP) via hyperpolarization [44]. The primary role of activated MC4R is to promote satiety and energy use; therefore, when depleted, it causes increased food intake, leading to obesity [45]. Multiple mice studies found that deletion of the MC4R locus produces a significant dysregulation of energy homeostasis, leading to obesity [44]. MC4R is directly stimulated by alpha-MSH, originally cleaved from its precursor peptide POMC. Several individuals suffer from a deficiency in POMC or α-MSH, leading to a lack of MC4R functioning and obesity. Mutations in these hormones in the melanocortin pathway led to the trial of testing the potency of MC4R agonists [46].

Setmelanotide, a potent MC4R agonist, has been shown to cause weight loss in patients with POMC or leptin receptor (LEPR) [47,48]. In single-arm, open-label, multicenter, phase 3 trials, subcutaneous once-daily setmelanotide was evaluated for efficacy and safety in individuals with severe obesity due to either POMC deficiency or LEPR deficiency [49]. Setmelanotide was injected daily, with a starting dose of 1.0 mg for adults and 0.5 mg for children. The dose is increased by 0.5 mg every two weeks until an individualized therapeutic dose is reached, up to a maximum of 3.0 mg. The therapeutic dose is a weight loss of approximately 2-3 kg per week for adults or 1-2 kg per week for children.

Results showed that 80% of POMC trial participants and 45% of LEPR trial participants achieved significant weight loss with at least a 10% decrease in body weight after approximately one year [49]. Additionally, decreased appetite was observed in phases 2 and 3 of clinical trials [50]. Setmelanotide was initially FDA-approved in 2020 for chronic weight management in individuals six years or older with obesity caused by POMC deficiency, proprotein subtilisin/kexin type 1 (PCSK1), or LEPR deficiency [46]. In 2022, setmelanotide became FDA-approved for individuals with Bardet-Biedl syndrome, a rare disorder characterized by central obesity, hypogonadism, and multiple organ dysfunction. However, it is not approved for treating obesity in individuals not impacted by POMC, PCSK1, LEPR deficiency, or Bardet-Biedl syndrome [46]. Although MC4R mutations are the leading monogenic cause of obesity, generating MC4R agonists for general obesity treatment has been challenging due to MC4R's location and function in the CNS. An increase in sympathetic nervous system effects is a major potential side effect for MC4R agonists that might outweigh the benefits of treating obesity [51].

Despite potential sympathetic adverse effects, one MC4R agonist emerging therapy undergoing clinical trials for treating generalized non-diseased obesity called RM-493 (Clinicaltrials.gov ID: NCT02041195; Clinicaltrials.gov ID: NCT01749137). It is also being evaluated in a phase 2 study for weight loss and hyperphagia in individuals with Prader-Willi syndrome (Clinicaltrials.gov ID: NCT02311673). Table 4 describes the up-to-date information about FDA-approval, drug dosing, indications, contraindications, and serious adverse effects of MC4R agonists.

**Table 4:** MC4R agonists as AOMs: FDA-approval, indications, contraindications, and serious adverse effects BBS: Bardet-Biedl syndrome

Drug	Formulation/dosing	Contraindications	Possible serious adverse effects	FDA-approval (year and indications)
					Setmelanotide (Imcivree^®^)	Recommended starting dosage injected subcutaneously for (1) Adults and pediatric patients aged 12 years and older is 2 mg (0.2 mL) once daily for two weeks. (2) Pediatric patients aged 6 to less than 12 years are 1 mg (0.1 mL) once daily for two weeks. Recommended target dosage for adults and pediatric patients aged 6 years and older is 3 mg (0.3 mL) injected subcutaneously once daily	Obesity due to suspected POMC, PCSK1, or LEPR deficiency not confirmed by genetic testing or with benign or likely benign genetic testing results	Male and female sexual function problems	2020
Other types of obesity that are not related to POMC, PCSK1, LEPR deficiency, or BBS, including obesity associated with other genetic conditions and general obesity	Depression and suicidal thoughts/actions	First and only FDA-approved treatment to target an impaired MC4R pathway, a fundamental cause of hunger and obesity in individuals with BBS, or POMC, PCSK1, or LEPR deficiency				
	Increased skin pigmentation					
	Darkening of skin lesions					
	Benzy alcohol toxicity					

Challenges associated with AOMs

AOMs have been found to be effective for weight loss, but their effectiveness can vary from person to person. While some people may experience significant weight loss, others may have little to no response. It is also unclear whether these medications can help maintain weight loss over the long term or if stopping them can reverse the benefits of weight loss. The long-term effects of AOMs are still not fully understood for newly approved medications, which is why it is crucial to evaluate the benefits versus risks before prescribing them to a patient. For instance, the recently approved drug semaglutide (Wegovy®) may cause serious side effects such as possible thyroid tumors (including cancer), pancreatitis, kidney problems, depression, or thoughts of suicide. Additionally, the cost of these medications can be high, and not all health insurance plans may cover them. Therefore, people who use AOMs should also make healthy lifestyle changes such as eating well and being active. However, it is important to note that these medications are not suitable for everyone, nor are they a cure for obesity. Obesity is a complex condition that requires a comprehensive approach to address its causes and consequences.

Despite the challenges associated with AOMs, patients with obesity-related health problems need to consult their physician and find the right medication to reduce their risk of comorbidities, such as cardiovascular diseases, and improve their mental and physical health. In addition, as more AOMs become available, physicians must stay informed of the advantages and disadvantages of these medications within their respective fields.

Discussion

AOMs are proven to be successful aids in weight loss alongside consistent healthy diet and exercise modifications. These medications are often prescribed when individuals are unsuccessful and finding difficulty losing weight through diet and exercise alone due to factors such as hormonal imbalances or genetics. Suppose a person cannot achieve weight loss through these methods; in that case, a healthcare professional may prescribe AOMs to help reduce the risk of developing other severe comorbidities associated with being obese, such as hypertension, hyperlipidemia, and diabetes. While it is important to target obesity directly, it is also important to address and prevent the various complications that may arise from obesity. Obesity predisposes to metabolic syndromes, such as hypertension, hyperlipidemia, and T2DM, increasing the risk for more serious illnesses, such as chronic kidney disease, heart failure, acute coronary syndrome, and fatty liver disease. Thus, targeting obesity before the development of comorbidities is a preventive measure. Many AOMs are available; finding the right one for an individual can involve trial and error. Some people may succeed with the first medication, while others may need to try different medicines to find one that works for them with minimal side effects.

Lipase inhibitors, cetilistat, and orlistat work by decreasing fat absorption and inhibiting fat hydrolysis. GLP-1 receptor agonists, such as liraglutide and semaglutide, stimulate insulin secretion, inhibit glucagon secretion, and delay gastric emptying, serving as appetite suppressants. Semaglutide (Wegovy®) is more effective than other GLP-1 receptor agonists in weight loss and is currently one of the most popular medications on the market. Leptin's role in decreasing food intake and increasing energy expenditure makes it important in weight maintenance. Its tendency for resistance limits it, but it is a valuable target for patients with obesity related to leptin deficiency, hence the significance of metreleptin. MC4R's appetite-reducing effect, secondary to the promotion of satiety and energy use, makes it an ideal therapeutic target; setmelanotide is especially beneficial for individuals with obesity related to POMC or MSH deficiency. Although metreleptin and setmelanotide are contraindicated in patients with generalized obesity, they show promise in treating obesity in patients with genetic causes that limit them from managing their weight solely with diet and exercise.

Beyond AOMs, there is the option for bariatric surgery. The indications for bariatric surgery are as follows: failure with diet and exercise modifications or refractory to AOMs and a BMI over 35 with comorbidities (T2DM, gastroesophageal reflux disease) or a BMI over 40 with or without comorbidities. Therefore, if a patient is refractory to AOMs but has a BMI less than 35, they will not qualify for bariatric surgery. Some patients with a BMI over 35 may skip AOMs altogether and receive bariatric surgery if they do not desire to take medications, which is acceptable.

## Conclusions

The various mechanisms of AOMs have been successful with diet modifications and exercise for weight loss. Lipase inhibitors and GLP-1 receptor agonists are the preferred medications for treating obesity, not secondary to a genetic cause that limits one’s ability to lose weight with diet and exercise alone. Leptin analogs and MC4R agonists are exclusively used for patients with obesity secondary to congenital/acquired generalized lipodystrophy and POMC deficiency, respectively. The prevalence and severity of the obesity crisis have resulted in great strides in medicine and advanced research to provide the most effective and inclusive treatments for weight loss in individuals with obesity. The expanding range of pharmaceutical options available for managing obesity offers hope for a healthier future with fewer medical complications attributed to obesity.
